# Comparative transcriptome reveal the potential adaptive evolutionary genes in *Andrias davidianus*

**DOI:** 10.1186/s41065-018-0056-6

**Published:** 2018-02-20

**Authors:** Qiaomu Hu, Quanhe Wang, Yan Meng, Haifeng Tian, Hanbing Xiao

**Affiliations:** 1 Yangtze River Fisheries Research Institute, Chinese Academy of Fishery Sciences, Wuhan, Hubei 430223 China; 2 College of Life Science, Yangtze University, Jingzhou, 434025 China

**Keywords:** *Andrias davidianus*, Comparative transcriptome, Evolution analysis, Adaptive genes

## Abstract

To search the evidence of molecular evolution mechanism for aquatic and cave habitat in *Andrias davidianus*, the evolution analysis was carried out among several species transcriptome data. The transcriptome data of *Notophthalmus viridescens*, *Xenopus tropicalis, Cynops pyrrhogaster, Hynobius chinensis* and *A. davidianus* were obtained from the Genbank and reassembled except *Xenopus tropicalis*. The BLAST search of transcriptome data obtained 1244 single-copy orthologous genes among five species*.* A phylogenetic tree showed *A. davidianus* to have the closest relationship to *H. chinensis*. Fourteen positively selected genes were detected in *A. davidianus* and *N. vridescens* group and fifteen in *A. davidianus* and *H. chinensis* group. Five genes were shared in the both groups which involved in the immune system, suggesting that *A. davidianus* adaptation to an aquatic and cave environment required rapid evolution of the immune system compared to *N. viridescens* and *H. chinensis*.

**Electronic supplementary material:**

The online version of this article (10.1186/s41065-018-0056-6) contains supplementary material, which is available to authorized users.

## Background

Amphibians played an important role as a transitional group linking aquatic to terrestrial in the evolution of vertebrates [1]. To elucidate evolutionary history, the genome and mitochondrial DNA are traditionally used to estimate divergence time [2]. Transcriptome sequencing has become a viable alternative to provide rapid developing genomic resources in non-model organisms [3, 4]. Comparative transcriptome analysis is used to estimate the non-synonymous substitution (Ka) and synonymous substitution (Ks) rates to calculate the evolutionary rate [5, 6] and hence, to identify genes involved in environmental adaptation. Distribution of synonymous substitutions can be used to calculate the divergent time based on the coding sequence [2, 7].

The Chinese giant salamander *Andrias davidianus* is a typical urodele, and an important species both as a biological resource and with respect to its value as a living fossil [8]. The species was historically widespread in China, but environmental degradation and human killing have led to its severe decline in the wild. From 1980s, it is classified as endangered by the International Union for Conservation of Nature and Nature Resources. Because of its irreplaceable protection status and good taste, artificial propagation technology was studied and succeeded at the end of 1990s. Success of artificial propagation technology provided a value way to protect the wild resources. In wild, it is aquatic in all life stages and typically inhabits rocky crevices in banks of streams and lakes, as well as subterranean rivers. To identify genes possibly related to *A. davidianus* adaptation to its aquatic life history and to a cave habitat, transcriptome data of other amphibian species were obtained from GenBank, and comparative transcriptome analysis was carried out to detect genes positively selected for in evolution.

## Methods

### RNA extraction and sequencing

Total RNA was extracted from five ovaries and testes using Trizol reagent (Invitrogen, USA) according manufacturer’s instructions and treated with RNase-free DNase I (Takara, China) to remove the genomic DNA, respectively. After RNA quality and quantity test, RNA was broken into short fragment, and first-strand cDNA was synthesized, and then the sequencing adapter was added. The cDNA libraries were constructed and sequenced on the Illumina sequencing platform (Illumina HiSeq™ 2500). All raw reads, low quality sequences, and reads containing adaptor sequences were removed, and the clean reads were obtained.

### Identification of orthologues genes and phylogenetic analysis

Two gonad transcriptome data (SRR3308418 and SRR3308420) of *A. davidianus* were provided by my lab. To expand data of *A. davidianus,* transcriptome data of skin (SRX729810) and spleen (SRX729743) were obtained from NCBI database and reassembled with the gonad transcriptome data. Transcriptome data of *N. viridescens* from heart, lens, brain, eye, liver, lung, spleen, kidney, testis, and ovary (ERR108189), *C. pyrrhogaster* lens and neural retina (SRR1051839), *H. chinensis* whole body (SRR1042328) and *X. tropicalis* from genome sequencing (GCA_000004195) were also obtained. The unigenes were reassembled from the downloaded raw reads, except for *X. tropicalis*. The numbers of unigenes for each species is given in Table 1. BLASTN software was used to align sequences, with the cutoff E-value set at 1e-7 [9]. OrthoMCL software was applied to classify the gene family [10]. Orthologous genes were obtained, and Venn diagrams were used to obtain the gene number [11]. The orthologous genes were used to construct the phylogenetic tree by the NJ method with 1000 bootstrap replications.

**Table 1:** Results of the assembly for each study species

Species	Transcriptome	N50	GC %	Max Length bp	Min Length bp	CDS
*Xenopus tropicalis* (XT)	22,855	2418	45.21	94,440	114	22,718
*Cynops pyrrhogaste* (CP)	122,913	1596	44.61	18,379	201	49,986
*Notophthalmus viridescens* (NV)	31,998	392	44.48	9697	201	17,943
*Hynobius chinensis* (HC)	103,800	426	47.54	15,293	201	51,362
*Andrias davidianus* (AD)	85,868	1492	48.61	17,741	201	43,402

### Estimate of substitution rates among species

Form the orthologous gene, only one orthologous gene in other species was classed as single-copy orthologous by PERL package [12, 13]. The single-copy orthologous genes were identified to calculate the synonymous substitution rates (Ks) and non-synonymous rate (Ka). The amino acid sequences were aligned by Muscle software [14]. The aligned sequences were converted to corresponding nucleotide sequences. Synonymous substitution rates (Ks) and non-synonymous rates (Ka) were estimated between species pairs by sit model under Codeml program in PAML package [15]. The best threshold was set at 0.5 based on the Ka/Ks value according to previous reports [5, 6]. Value of two fold log-likelihood difference was used to perform a Chi-squared test and the difference of the parameter number was set as the degree in the Chi-squared. Positively selected sites were allowed when P was < 0.05 and posterior probability was > 0.95 [16]. A Ka/Ks value > 1 indicated strong positive selection, from 0.5 to 1 indicated weak positive selection, and a value < 0.1 indicated negative selection.

## Results

### Orthologue identification and phylogenetic analysis

**Fig. 1:**
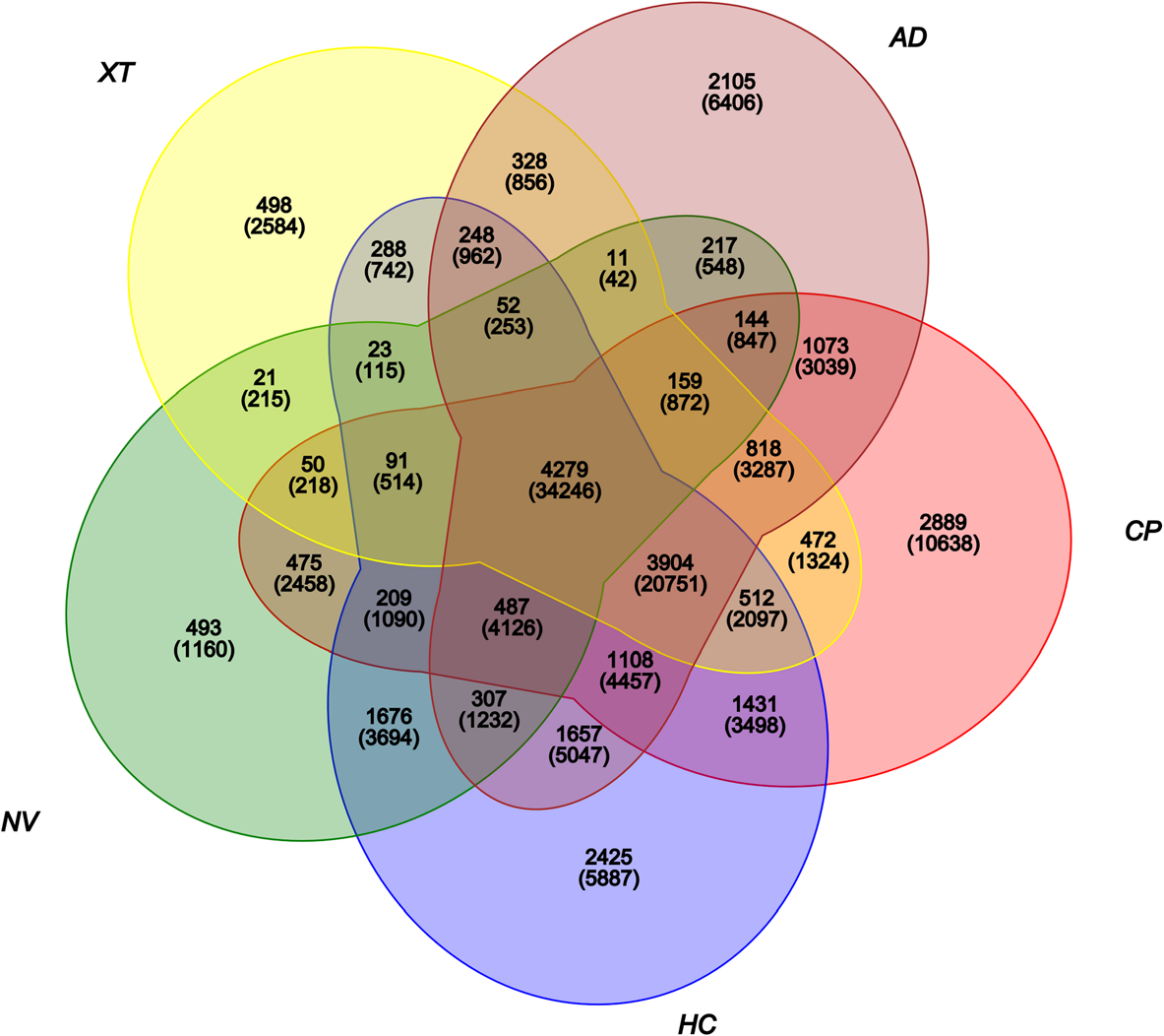
Venn diagrams showing the unigenes for comparative transcriptomes. The superscript indicates the protein family and the subscript indicates the unigenes

To identify the phylogenetic relationship among the species, large-scale transcriptome characterizations were carried out for *N. viridescens, X. tropicalis, C. pyrrhogaster, H. chinensis,* and *A. davidianus*, and transcriptome data were downloaded and reassembled (Table 1). Comparative analysis yielded 4279 gene families and 34,246 putative orthologous genes (Fig.1). To construct the phylogenetic tree with *X. tropicalis* as out-group, 1244 single-copy orthologous genes were identified. The phylogenetic tree showed *A. davidianus* to have the closest relationship to *H. chinensis*, with *N. viridescens* and *C. pyrrhogaster* clustered on one separate branch (Additional file 1: Figure S1).

### Evolutionary profile of *Andrias davidianus* genes

**Fig. 2:**
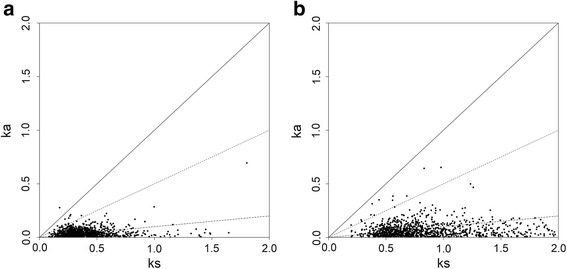
Ka/Ks ratio of 1244 single-copy orthologous genes. **a**. Ka/Ks distribution in *Andrias davidianus* and *Hynobius chinensis*, **b**. Ka/Ks distribution in *Andrias davidianus* and *Notophthalmus viridescens*. The solid line shows the threshold of Ka/Ks = 1, the dashed line marked the weak positive selection threshold of Ka/Ks = 0.5, and the short dashed line represented threshold of Ka/Ks = 0.1

We analyzed the evolutionary pattern of 1244 single-copy orthologous genes in *A. davidianus, H. chinensis, and N. viridescens.* Synonymous (Ks) and non-synonymous (Ka) substitutions per site were observed (Fig. 2). A majority of sequence pairs showed a Ka /Ks < 0.5, implying that these genes involved negative selection. Fifteen rapidly evolving sequences were identified with Ka/Ks > 0.5 between *A. davidianus* and *H. chinensis*, and 14 such sites were observed between *A. davidianus* and *N. viridescens* (Additional file 2: Table S1).

## Discussion

Next-generation sequencing technology yielded a large number of sequences at the low cost and provides more sequences compared to traditional sequencing methods [17, 18]. Due to the cost and the throughput, genome-wide detection of the adaptive evolution gene was performed in many species by next-generation sequencing [17, 19]. Comparative phylogenetic analysis at the genome level improved the precision of evolutionary inference compared to single gene [20]. However, because of the large genome of the *A. davidianus,* evolutionary analysis by comparative genome was hard to carry out. Transcriptome sequencing was a valuable way to obtain large-scale sequences without reference genome [21, 22]. Phylogenetic analysis of transcriptome sequence data exhibited high supported tree topologies in many species [23, 24].

To elucidate the phylogenetic evolution of *A. davidianus*, comparative transcriptome analysis was conducted to construct the phylogenetic tree with *X. tropicalis* as out-group. To search adaptive gene for aquatic and cave life, molecular evolution was analyzed among the related species. Synonymous substitution rates (Ks) and non-synonymous substitution rates (Ka) were calculated according to the phylogenetic tree by PAML software [15, 25], with the optimal threshold for selecting the positively expressed sequence tag (EST) of 0.5 based on previous study [25]. Several positively selected genes were detected. Similar results were found in topmouth culter *Erythroculter ilishaeformis* and zebrafish *Danio rerio*, in which 38 candidate genes exhibited signs of positive selection with dN/dS ratios > 0.5 [6]. Five genes related to the immune system [26–29] [cystatin-like, oncostatin-M-specific receptor subunit beta isoform X1(OSMF), exonuclease, cell death regulator Aven, and centromere protein H] were detected in the *A. davidianus*/*H. chinensis* and *A. davidianus*/*N. viridescens* groups. *Andrias davidianus* is aquatic and inhabiting subterranean rivers and caves while *N. viridescens* and *H. chinensis* are mainly terrestrial and only special stage in water.

Aquatic and cave dwelling organisms generally encounter more bacteria than do terrestrial animals. Thus, the *A. davidianus* immune system should show more rapid mutations, as was confirmed in our investigation. Due to lack of full-length according to the transcriptome sequencing, many gene relevant to positive selection was omitted and Ka/Ks ratio was decreased from normal level [6]. Further study will be carried out to identify the genes under positive selection.

## Additional files

Additional file 1: **Figure S1.** Phylogenetic tree of selected species based on 1244 single-copy orthologous genes. (TIFF 212 kb)

Additional file 2: **Table S1.** Orthologs gene under positive selection among species. (DOCX 18 kb)

## Abbreviations

EST: Expressed sequence tag
Ka: Non-synonymous substitution rates
Ks: Synonymous substitution rates
OSMF: Ooncostatin-M-specific receptor subunit beta isoform X1
