# Supplementary figures and images for: Comparative transcriptome reveal the potential adaptive evolutionary genes in Andrias davidianus
Source: Hereditas. 2018 Feb 20;155:18. doi: 10.1186/s41065-018-0056-6 (PMC5819198; doi:10.1186/s41065-018-0056-6)

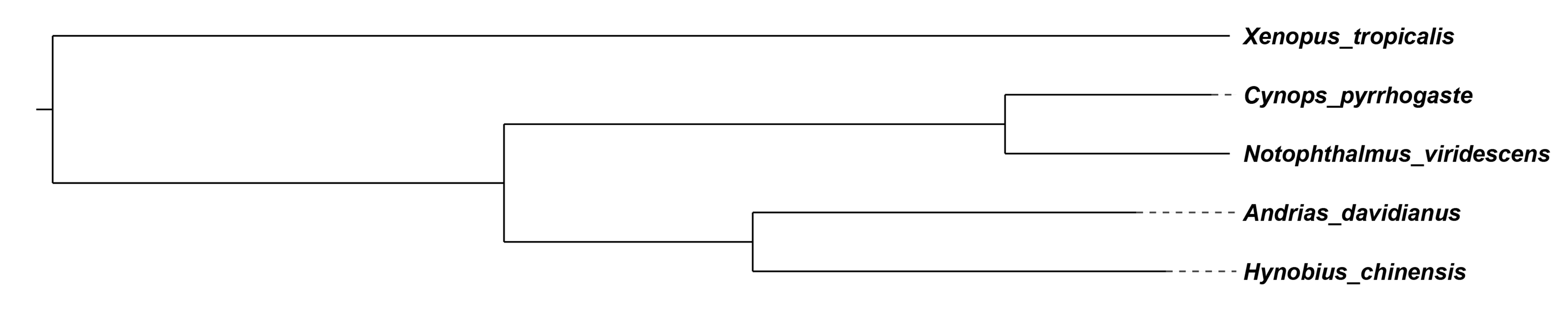

Supplement: Supplementary file 1 — Figure S1. Phylogenetic tree of selected species based on 1244 single-copy orthologous genes. (TIFF 212 kb) [file 41065_2018_56_MOESM1_ESM.tif]
