# Supplementary material for: Comparative transcriptome reveal the potential adaptive evolutionary genes in Andrias davidianus
Source: Hereditas. 2018 Feb 20;155:18. doi: 10.1186/s41065-018-0056-6 (PMC5819198; doi:10.1186/s41065-018-0056-6)
Supplement: Supplementary file 2 — Table S1. Orthologs gene under positive selection among species. (DOCX 18 kb) [file 41065_2018_56_MOESM2_ESM.docx]

**Comparative transcriptome reveal the potential adaptive evolutionary genes in Andrias davidianus**

Qiaomu Hu*^1^, Quanhe Wang^1, 2^, Yan Meng^1^, Haifeng Tian^1^, Hanbing Xiao^1^*

**Supplementary Information :**

Figure S1. Phylogenetic tree of selected species based on 1244 single-copy orthologous genes.

Table S1. Orthologs gene under positive selection among species.

|  | Gene ID | Ka/Ks | Gene accession | Annotation |
| --- | --- | --- | --- | --- |
| HC/AD | Unigene0016425 | 0.515 | XP_006009108.1 | probable palmitoyltransferase ZDHHC1-like |
|  | Unigene0033116 | 0.557 | XP_012396914.1 | ankycorbin isoform X1 |
|  | Unigene0082580 | 0.781 | XP_005287507.1 | cystatin-like ▲▲ |
|  | Unigene0025121 | 0.705 | NP_001039169.1 | novel protein similar to prothymosin, alpha |
|  | Unigene0011651 | 0.77 | KFV52470.1 | Nuclear fragile X mental retardation-interacting protein 2 |
|  | Unigene0025259 | 0.686 | XP_005282271.1 | oncostatin-M-specific receptor subunit beta isoform X1★★ |
|  | Unigene0016588 | 0.559 | KFV78550.1 | Thyroid transcription factor 1-associated protein 26 |
|  | Unigene0028123 | 0.501 | XP_008178007.1 | protein FAM178A |
|  | Unigene0010844 | 1.584 | NP_001006573.1 | exonuclease ☆☆ |
|  | Unigene0078114 | 0.565 | NP_001081203.1 | tyrosine phosphatase |
|  | Unigene0081968 | 0.54 | XP_007058938.1 | LOW QUALITY PROTEIN: glomulin |
|  | Unigene0085780 | 0.542 | XP_005286621.1 | bolA-like protein 3 |
|  | Unigene0018195 | 0.509 | XP_005377323.1 | synaptophysin-like protein 1 |
|  | Unigene0029880 | 0.753 | XP_006132743.1 | cell death regulator Aven ◇◇ |
|  | Unigene0026596 | 0.503 | XP_007059081.1 | centromere protein H ●● |
| NV/AD | Unigene0082580 | 0.566 | XP_005287507.1 | cystatin-like ▲▲ |
|  | Unigene0071170 | 0.572 | XP_005293057.1 | LOW QUALITY PROTEIN: caspase-9 |
|  | Unigene0017551 | 0.579 | NP_001107377.1 | Williams-Beuren syndrome chromosomal region 27 protein |
|  | Unigene0046340 | 0.773 | XP_007070061.1 | syndecan-1 |
|  | Unigene0051088 | 0.521 | XP_007065365.1 | nudC domain-containing protein 1 |
|  | Unigene0024446 | 0.585 | XP_005310930.1 | thymidine kinase 2, mitochondrial isoform X1 ★★ |
|  | Unigene0025259 | 0.62 | XP_005282271.1 | oncostatin-M-specific receptor subunit beta isoform X1 |
|  | Unigene0017652 | 0.596 | XP_010571475.1 | protein Jumonji isoform X2 |
|  | Unigene0010844 | 0.52 | NP_001006573.1 | exonuclease☆☆ |
|  | Unigene0010186 | 0.686 | XP_007064660.1 | ETS translocation variant 3 |
|  | Unigene0084425 | 0.796 | NP_001120210.1 | adrenodoxin-like protein, mitochondrial |
|  | Unigene0069494 | 0.539 | XP_009577555.1 | LOW QUALITY PROTEIN: rho GTPase-activating protein 31-like |
|  | Unigene0029880 | 0.666 | XP_006132743.1 | cell death regulator Aven ◇◇ |
|  | Unigene0026596 | 0.824 | XP_007059081.1 | centromere protein H ●● |

NV = *Notophthalmus viridescens*, HC = *Hynobius chinensis* and AD = *Andrias* *davidianus*. Identical symbols indicate same gene shared between the two groups and quantity represent number.
